# The Efficacy of Carbon Dioxide Paste in Alleviating Pain in Patients After Neck Dissection: Protocol for a Double-Blinded, Randomized Controlled Trial

**DOI:** 10.2196/50500

**Published:** 2023-11-13

**Authors:** Yoshiaki Tadokoro, Daisuke Takeda, Izumi Saito, Nanae Yatagai, Yasumasa Kakei, Masaya Akashi, Takumi Hasegawa

**Affiliations:** 1 Department of Oral and Maxillofacial Surgery, Kobe University Graduate School of Medicine Kobe Japan

**Keywords:** carbon dioxide, double-blind, neck dissection, pain, paste, postoperative, randomized controlled trial, surgery, surgical

## Abstract

**Background:**

Head and neck cancers that cause severe aesthetic and functional disorders normally metastasize to the cervical lymph nodes. Patients with cervical lymph node metastasis are undergoing neck dissection. Shoulder complaints are common after neck dissection, with patients reporting symptoms such as pain, weakness, shoulder droop, and disability. However, no safe and effective treatment is available for this condition at present. We will conduct a double-blinded, randomized controlled trial to evaluate the efficacy of carbon dioxide (CO_2_) paste in relieving pain in patients after neck dissection.

**Objective:**

This will be the first clinical study to compare the efficacy of CO_2_ paste with placebo in relieving postoperative pain in patients who underwent neck dissection.

**Methods:**

We will perform this trial at the Kobe University Hospital in Japan. Patients will be randomized 1:1 into the CO_2_ paste and control groups. Patients in the CO_2_ paste group will have the CO_2_ paste applied to the cervical surface skin for 10 minutes once per day for 14 consecutive days. The primary end point of the study is a change in the visual analog scale (VAS) scores of neck pain from baseline on day 1 (preapplication) to the end of drug application (day 15). Secondary end points include changes in the following parameters from baseline on day 1 to the end of drug application (day 15) or the study (day 29): neck pain VAS score (days 1-29), grip strength (days 1-15 and 1-29), VAS scores for subjective symptoms (the feeling of strangulation, numbness, swelling, and warmth in the neck and shoulder region) for days 1-15 and 1-29, whether the VAS score improved more than 30% (days 1-15), the arm abduction test (days 1-15 and 1-29), shoulder range of motion (abduction and flexion) for days 1-15 and 1-29, occurrence of skin disorders, and occurrence of serious side effects. Periodic monitoring will be conducted for participants during the trial. This study was approved by the certified review board of Kobe University.

**Results:**

The intervention commenced in May 2021 and will continue until March 2024. The collected data will provide information on the efficacy of the CO_2_ paste treatment. The primary end point will be compared using the Wilcoxon test, with the 1-sided significance level set at 5%. Each evaluation item will be summarized. Secondary efficacy end points will be analyzed to provide additional insights into the primary analysis. Findings based on the treatment effects are expected to be submitted for publication in 2025.

**Conclusions:**

This trial will provide exploratory evidence of the efficacy and safety of CO_2_ paste in relieving pain in patients after neck dissection.

**Trial Registration:**

Japan Registry of Clinical Trials (jRCT) identifier: jRCTs051210028; https://jrct.niph.go.jp/en-latest-detail/jRCTs051210028

**International Registered Report Identifier (IRRID):**

DERR1-10.2196/50500

## Introduction

Postoperative wound pain in the head and neck region generally improves within 1-2 weeks after surgery. However, patients undergoing cancer treatment often experience continuous, persistent pain, which can cause physical and psychological distress. Surgery and radiation therapy often cause painful scarring and contractures. Musculoskeletal and neuromuscular disabilities after radiotherapy and surgical treatment are major causes of cancer treatment–related side effects. Disabilities in the head and neck region after cancer treatment are related to a patient’s ability to communicate, eat, and work [1]. These changes reduce the functioning and social interaction of these patients [1,2].

Shoulder complaints are common (18%-77%) after neck dissection, even if the spinal accessory nerve has been preserved [3,4]. Symptoms such as pain, weakness, shoulder droop, and disability have been reported in patients after neck dissection [5,6]. Pain is an important factor affecting the quality of life (QOL) of cancer survivors [1,7]. Although various drug treatments are available for preventing or improving the management of chronic postsurgical pain [8-10], these treatments have side effects.

The exact etiology of persistent postsurgical pain remains unclear, but its causes are thought to involve neuropathy [11-13], inflammation [14,15], central sensitization [16,17], and ischemia [18-20]. In rats, decreased blood flow and tissue ischemia cause complex regional pain syndrome–like symptoms [18]. In addition, patients with peripheral arterial diseases experience chronic ischemic pain [20]. Thus, continuous postoperative pain may be reduced by improving the blood flow in the ischemic scar tissue.

We had previously reported the effects of transcutaneous application of carbon dioxide (CO_2_) in a hydrogel as an absorption-promoting agent in healthy individuals and various animal models [21-23]. Transcutaneous application of CO_2_ improves vascularization and exercise performance in rats; it also increases local oxygenation, causing the dissociation of oxygen from hemoglobin (Bohr effect) [23-25]. However, this method uses CO_2_ gas, which is difficult to apply to the head and neck. In a recent study, we investigated the usefulness, safety, and feasibility of a CO_2_ paste by analyzing the changes in blood flow in a phase 1 clinical study in healthy individuals [26]. The CO_2_ paste reacted with water on the skin surface, could infiltrate CO_2_ transcutaneously without the use of external CO_2_ gas, and could be applied to the head and neck region. In the phase 1 clinical study, application of the CO_2_ paste increased the blood flow in the skin and muscles, as observed by dynamic magnetic resonance imaging, and caused no adverse reactions.

This double-blind, randomized controlled trial aims to evaluate the efficacy and safety of CO_2_ paste in decreasing postoperative neck pain in patients undergoing neck dissection.

## Methods

### Objectives

This study will examine whether the application of a CO_2_ paste can reduce postoperative neck pain in patients receiving neck dissection in a double-blinded, randomized controlled trial. The primary objective is to estimate the within-patient differences in visual analog scale (VAS) scores for neck pain ranging from 0 (no pain) to 10 (worst pain) from baseline (day 1: before application) to the end of the study drug application (day 15). The secondary objectives are to evaluate the within-patient differences in the VAS scores for neck pain from baseline (day 1: before application) to the end of study drug application (day 15), grip strength from baseline to the end of the drug application (days 1-15) and the end of the study period (days 1-29), VAS scores for subjective symptoms (the feeling of strangulation, numbness, swelling, and warmth) from baseline to the end of the drug application (days 1-15) and the end of the study (days 1-29), arm abduction [27] from baseline to the end of the drug application (days 1-15) and the end of the study (days 1-29), and shoulder joint range of motion (abduction or flexion) from baseline to the end of the drug application (days 1-15) and the end of the clinical study (days 1-29). In addition, we aim to evaluate whether patients showed a ≥30% improvement in the VAS scores for neck pain from baseline to the end of the drug application (days 1-15) and the end of the study (days 1-29).

### Study Design

**Figure 1:**
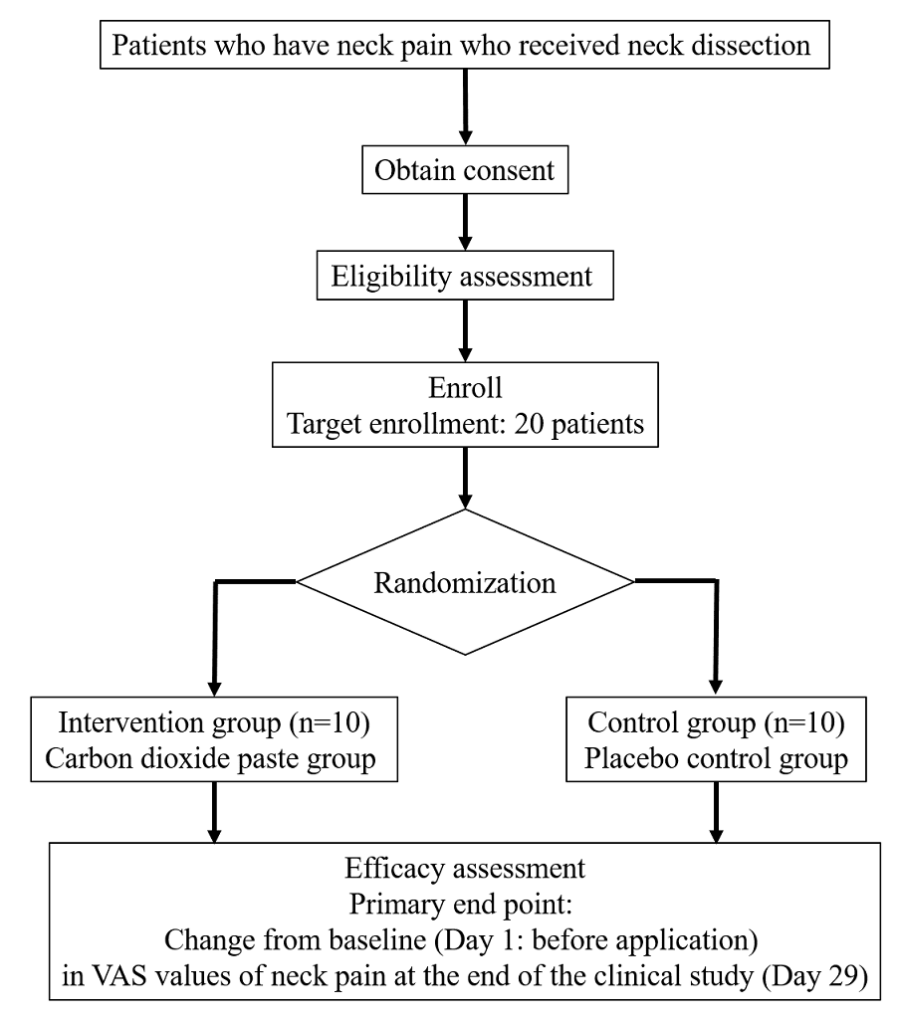
Flowchart of participants. VAS: visual analog scale.

This study has been designed as a single-center, placebo-controlled, double-blind, randomized controlled trial. The patient flowchart is shown in Figure 1. This study will be conducted at Kobe University Hospital. All data will be stored and archived at the data center of Kobe University Hospital. We will use Research Electronic Data Capture (REDCap; Vanderbilt University), an electronic data system for clinical research, to manage the data and protect confidentiality before, during, and after the trial. The study population will consist of patients undergoing neck dissection and will be selected based on the inclusion and exclusion criteria listed in Textbox 1. Patients will be recruited from Kobe University Hospital from May 24, 2021, to March 31, 2024. All patients will be asked if they wish to participate in the study and will be enrolled after obtaining written informed consent.

**Inclusion and exclusion criteria for the study.**

**Inclusion criteria**

- Aged 20 years or older
- Have complaints of postoperative neck pain after unilateral cervical dissection
- Between 6 months and 3 years after neck dissection
- A visual analog scale score of 1 or higher for neck pain
- Have given voluntary consent to participate in this clinical study

**Exclusion criteria**

- Hypersensitivity to drugs, including severe allergies
- Peripheral arterial disease, ischemia (Fontaine III-IV), and ischemic cerebral artery disease
- Heart failure (New York Heart Association IV) and ischemic heart disease (Canadian Cardiovascular Society functional classification 3-4)
- Female patients who are pregnant, lactating, wishing to become pregnant during the study period, or are fertile and cannot accept an effective contraceptive method
- Severe respiratory disease with oxygen saturation (SpO2) less than 90% at rest
- Skin ulcers larger than 4 cm × 4 cm × 1 cm or skin diseases making it difficult to apply the paste
- Deemed inappropriate for participation in this clinical study by the investigators

### Randomization (Allocation)

Using the permutation random block method stratified by category, participants will be randomly assigned to either the CO_2_ paste group or the placebo control group at a 1:1 allocation ratio. To ensure that blinding is maintained, the block size will not be disclosed. The allocation sequence for the randomization method will be generated by a biostatistician. All participants who provide consent to participate, fulfill the inclusion criteria, and do not meet any of the exclusion criteria will be randomized. The eligibility of each participant will be confirmed. A subject-enrollment confirmation form containing the eligibility judgment, randomization assignment results from the generated random sequence, and the enrollment number will be issued.

### Experimental Drugs

The CO_2_ paste (direCO_2_t) is manufactured as an experimental drug at the CO2TECH, a limited liability company. The CO_2_ paste contains 1,3-butylene glycol and other components, including sodium hydrogen carbonate, malic acid, sodium dihydrogen phosphate, an alkyl-modified carboxyvinyl polymer, and carboxyvinyl polymer. The placebo paste is a formulation in which the sodium bicarbonate in the CO_2_ paste is replaced by a nonreactive material (sodium chloride).

### Treatment and Assessment Schedule

Within 4 weeks of enrollment, the VAS score for neck pain will be evaluated 1 week before the administration of the experimental drug during the preobservation period. On the first day of treatment, body temperature, blood pressure, oxygen saturation, and pulse rate will be measured by a physician, dentist, or nurse before and after the application of the experimental drug. The paste will be applied at 0.2 g-0.3 g/100 cm^2^ to the affected neck area once per day for 14 consecutive days. The duration of each application will be 10 minutes, and the paste will be wiped off after application.

### Data Collection

The data collection schedules are presented in Table 1. The primary investigator or subinvestigator will enter the case report form (CRF) data for each participant into the electronic data capture (EDC) system. The principal investigator will confirm that the entered CRF data are complete and correct, electronically sign the CRF on the EDC system, and print out the signed CRF for filing. The CRF printout will be retained. If any queries about the CRF data are entered by the staff at the data center, the primary investigator or subinvestigator will be required to promptly respond to them.

**Table 1:** Data collection schedule.

	Registration (within 4 weeks before allocation)	Preobservation (7±3 days before allocation)	Allocation day 1 (preapplication)	Allocation day 1 (postapplication)	Postallocation day 15+1 (finish application)	Postallocation day 29+3	Exclusion	Within 7 days after exclusion
Assessment of eligibility criteria	✓							
Obtain consent	✓							
Registration	✓							
Patient characteristics^a^	✓							
Allocation and start of the application			✓					
VAS^b^ of neck pain		✓	✓	✓	✓	✓		
VAS values of subjective symptoms^c^			✓	✓	✓	✓		
Strength of grip arm abduction test^d^ Shoulder joint range of motion (abduction or flexion)			✓		✓	✓		
Adverse events				✓	✓	✓	✓	✓
Vital sign^e^	✓		✓	✓	✓		✓	✓
Oxygen saturation (SpO_2_)	✓		✓				✓	✓
Confirmation of days of application					✓		✓	

^a^Sex, date of birth, age, medical history, comorbidity, primary disease, TNM classification, the number of days after surgery, the range of neck dissection, preservation of sternocleidomastoid muscle, accessory nerve, and internal jugular vein, and the details of adjuvant therapy.

^b^VAS: visual analog scale.

^c^Strangulation, numbness, swelling, and warmth.

^d^Arm abduction test: up to 180˚ without pain or effort (5 points), up to 180˚ but with pain or effort (4 points), up to more than 150˚ but less than 180˚ (3 points), up to more than 90˚ but not less than 150˚ (2 points), up to approximately 90˚ (1 point), and up to less than 90˚ (0 point) [27].

^e^Body temperature, blood pressure, and pulse rate.

### Main Outcomes

The primary end point for efficacy is the change in the VAS score for neck pain from baseline (day 1: before application) to the end of the study drug application (day 15).

The secondary efficacy end points are as follows:

1. Change in the VAS score for neck pain from baseline (day 1: before application) to the end of the clinical study (day 29).
2. Changes in grip strength from baseline to the end of the drug application (days 1-15) and the end of the study (days 1-29).
3. Changes in VAS scores for subjective symptoms (feeling of strangulation, numbness, swelling, and warmth) from baseline to the end of the drug application (days 1-15) and the end of the study (days 1-29).
4. Whether the VAS score for neck pain showed a ≥30% improvement from baseline to the end of the drug application (days 1-15) and the end of the study (days 1-29).
5. Change in arm abduction [27] from baseline to the end of drug administration (days 1-15) and the end of the study (days 1-29).
6. Change in shoulder joint range of motion (abduction and flexion) from baseline to the end of drug application (days 1-15) and the end of the study (days 1-29).

The secondary end point for safety is the presence or absence of adverse events such as skin disorders and serious diseases. The relationships among the interventions, outcomes, other assessments, and visits associated with the participants in this study are shown in Table 1.

### Statistical Analysis

The primary objective of this clinical study was to evaluate the efficacy of the CO_2_ paste versus the placebo in terms of the change in VAS scores for neck pain (the primary end point). For the comparison of the primary end point, the Wilcoxon test will be performed for the null hypothesis, “There is no difference in the change in VAS values for neck pain at day 15 between the placebo group and the CO_2_ paste group.” The significance level will be set at 5.0% (1-sided). Each evaluation item will be summarized. Secondary efficacy end points will be analyzed to provide additional insights into the primary analysis. No adjustment for multiplicity will be made in the analyses of the secondary efficacy end points. The significance level for hypothesis testing is 5% (2-sided) with 2-sided 95% CIs. The incidence and rate of disease will also be evaluated. The safety end point is the frequency of the occurrence of adverse reactions. Tables will be prepared for the end points, and exact 2-sided 95% CIs of the binomial distribution will be calculated for each group to estimate the proportions.

### Sample Size Calculation

Most patients are expected to have a baseline VAS score of 2-3 cm. For the primary end point (the amount of change in VAS values for neck pain), we estimated that the mean difference in change between the CO_2_ paste application and placebo groups would be 2 (SD 1.5) cm. A Wilcoxon test (a nonparametric test) will be performed considering the possibility that participants with a baseline score of less than 2 cm might be enrolled. The number of cases based on the Wilcoxon test can be calculated by multiplying the number of cases based on the *t* test [28]. Although the constant for multiplication is 1.045 in the case of a normal distribution, since the participants in this study were not assumed to be normally distributed, the number of cases was calculated by multiplying by 1.2. When conducting a *t* test with a 1-sided significance level of 5.0% and a power of 80%, a total of 7 participants were required per group. When the number of cases was multiplied by 1.2, the total number of cases was 8.4. Therefore, considering drop out cases, 20 cases would be sufficient to maintain power.

### Monitoring for Human Rights, Safety, and Adequacy

Monitoring will be conducted periodically to check whether the human rights and safety of the participants are protected and to confirm that the regulatory requirements of the Clinical Trials Act are followed. To ensure an appropriate assessment, the principal investigator is responsible for dealing with personal data and items specified in the written procedure. Any requirements made by the ethics committee will be immediately announced to the participants by the study investigators.

### Other Considerations

This study will be conducted without patient or public involvement. Neither the patients nor the public will be involved in the development of the research question, study design, or implementation of the trial. Patients will not be invited to develop patient-relevant outcomes, interpret the results, or participate in the writing or editing of the final manuscript. Since the interventions in this study are routine clinical procedures, the burden of the interventions will be assessed by the patients themselves.

Any protocol changes that affect the study conduct or participant risk-benefit profile will require approval from the relevant institutional review board, including changes in the objectives, design, sample size, participant characteristics, staff changes, or significant administrative features. Minor protocol corrections or clarifications that do not affect the study conduct or the participant risk-benefit profile will be viewed as administrative changes and documented internally. The study investigators will have full access to and ownership of all the data. Anonymized data will be made available to other interested investigators for additional analyses on reasonable request, following reports of primary outcomes and with an appropriate data use agreement. The findings of this study will be disseminated through scientific and professional conferences and a peer-reviewed journal.

### Ethical Considerations

The study is being conducted in compliance with the principles of the Declaration of Helsinki (1996), the principles of good clinical practice, and all applicable regulatory requirements. This study has been approved by the Clinical Research Review Board of Kobe University (CRB5180009), and the trial has been registered with the Japan Registry of Clinical Trials (jRCT) (jRCTs051210028). All participants will be required to provide written informed consent before any study procedures are conducted. The participants will have the opportunity to review the consent form and confirm that they have fully understood the details of the study procedures. Secondary use of the data will occur only if the patients provide written informed consent for additional use of their data. Secondary use of the data will adhere to appropriate ethical review and approvals, as per institutional guidelines. No funding was received for this study, including institutional or departmental support. The study is not commissioned, externally peer reviewed.

## Results

The subject recruitment began on May 24, 2021. The entry period for participants is up to March 31, 2024, three years after the jRCT registration. Data analyses of all enrolled participants will be conducted in 2024. This manuscript is based on the current version of the protocol (version 3.0), last updated on December 23, 2022.

The data sets generated in this study will be available on the jRCT website. The final report is expected to be published in a peer-reviewed journal in 2025. The findings will be disseminated through scientific and professional conferences and peer-reviewed journal publications in 2025.

## Discussion

### Expected Findings and Implications

This report describes the protocol for a study evaluating the application of CO_2_ paste to patients who undergo neck dissection and experience postoperative complaints. A neck dissection is a surgical procedure performed to remove cervical lymph nodes. During surgery, the blood vessels are ligated to prevent bleeding. In addition, the internal jugular vein, which drains blood from important organs such as the brain, face, and neck, is removed depending on the type of neck dissection. These blood flow interruptions lead to wound ischemia and various postoperative symptoms or dysfunctions.

Pain, the main side effect of neck dissection, often aggravates patient QOL [1,7]. As its pathogenesis remains uncertain, a treatment strategy for postoperative pain has not yet been established. Analgesics such as nonsteroidal anti-inflammatory drugs and acetaminophen are commonly administered to relieve this pain. Nonsteroidal anti-inflammatory drugs show remarkable efficacy as painkillers; however, they are also associated with serious adverse reactions in the gastric mucosa and cardiovascular, hepatic, renal, and hematological systems [29]. Although almost all patients receive acetaminophen [30], those undergoing neck dissection continue to experience pain. Considering the limitations of drug therapy, effective treatment of every patient with postoperative pain is essential.

In a phase 1 clinical study, we reported that CO_2_ paste application increases blood flow to the skin and muscles without any side effects [26]. Since transcutaneous CO_2_ application induces angiogenesis by inducing an artificial Bohr effect, we hypothesize that this simple and adaptable treatment can alleviate side effects and improve the QOL of patients undergoing neck dissection.

This study will be the first to apply CO_2_ paste to postoperative skin. Since it is composed of food ingredients, the CO_2_ paste is considered to have few life-threatening side effects. However, there is no evidence on whether its application may induce dermatitis. Therefore, the occurrence of dermopathies such as erythema and exanthema should be carefully monitored during the trial. To address potential safety concerns, the CO_2_ paste will be applied to the postoperative neck for only 10 minutes a day.

Through this study, we aim to determine the curative effect of CO_2_ paste application on postoperative neck pain in patients undergoing neck dissection. Since the efficacy of CO_2_ paste for neck pain has never been reported, this will be the first well-designed clinical evaluation study on this topic. Our primary hypothesis is that CO_2_ paste application can reduce pain in patients undergoing neck dissection. We also expect that the application of this paste will reduce subjective symptoms, including feelings of strangulation, numbness, swelling, and warmth, and will improve grip strength, arm abduction, and shoulder joint range of motion (abduction and flexion).

### Study Strengths and Limitations

We previously conducted a clinical trial on a small number of healthy individuals. To explore the adequacy and applicability of CO_2_ paste application, this will be the first protocol to be performed as a phase 2, double-blinded, randomized controlled trial, evaluating the efficacy of CO_2_ paste in a small number of patients with neck pain after neck dissection. Nonetheless, this clinical trial has some limitations that should be acknowledged. First, the number of evaluated patients is relatively small to conclusively determine the efficacy of the CO_2_ paste. Second, since some patients underwent neck dissection as part of larger resections, a small amount of heterogeneity is expected. Finally, this study is limited by its short duration (28 days).

### Conclusions

Considering the general condition of the patients and the side effects of drug therapy, postoperative pain after neck dissection is difficult to manage. Therefore, establishing a widely applicable treatment with fewer adverse effects is essential. The results of this study will provide valuable evidence for the future application of CO_2_ paste to patients complaining of pain after neck dissection.

## Abbreviations

CO_2_: carbon dioxide
CRF: case report form
EDC: electronic data capture
jRCT: Japan Registry of Clinical Trials
QOL: quality of life
REDCap: Research Electronic Data Capture
VAS: visual analog scale

## Data Availability

The data sets generated or analyzed during this study are available from the corresponding author on reasonable request.
